# Editorial: Chemistry, a Sustainable Bridge From Waste to Materials for Energy and Environment

**DOI:** 10.3389/fchem.2020.641129

**Published:** 2021-01-27

**Authors:** Francesca Deganello, Ana C. Tavares, Enrico Traversa

**Affiliations:** ^1^ Istituto per lo Studio dei Materiali Nanostrutturati, Consiglio Nazionale delle Ricerche Italiano (CNR), Rome, Italy; ^2^ Centre Énergie Matériaux Télécommunications, Institut National de la Recherche Scientifique, Varennes, QC, Canada; ^3^ School of Materials and Energy, University of Electronic Science and Technology of China, Chengdu, China

**Keywords:** waste—a misplaced resource, materials from waste, environment and energy application, chemistry, sustainable synthesis

The recent acceleration of climate changes and associated catastrophic phenomena worldwide is calling for unprecedented efforts for protecting our environment. The United Nations Sustainable Development Goals (SDGs) are now playing a major role in the policies of many governments. One of the main tools for reaching sustainability is adopting the concept of circular economy. We cannot afford anymore the unlimited growth of waste as in the linear economy model, but we should go toward reusing and recycling of commodities at their end of life cycle. In this framework, the transition to sustainability needs new sustainable approaches in chemical synthesis, since chemistry has a predominant role in the production of new materials.

The present research topic focuses on the possibility to use waste-derived precursors for preparing new materials (Figure 1) for environmental remediation or for sustainable energy applications. The general idea is based on the sustainability concepts, which consider both the limitation of a damage and the creation of a benefit. Thus, the use of waste, which needs to be eliminated, often at some cost, could be seen as a damage limitation. The production of materials from waste-precursors is considered benefic for the society and economy. If the material is used for environmental and energy applications, benefit is further increased, and damage is further limited.

**FIGURE 1:**
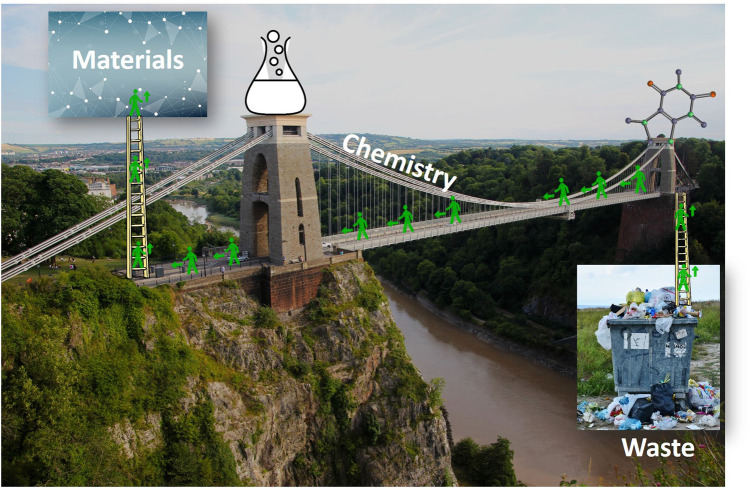
In this Research Topic, Chemistry is presented as a bridge connecting waste to materials, that can be further used for a greener and sustainable world.

The majority of the research work in this field still concerns organic waste, which can be transformed into carbon-based materials to be used in the formulation of heterogeneous catalysts and electrochemical double-layer capacitors or as pollutant absorbents. For example, the lignin fraction of the pitch pine sawdust was extracted and transformed into a porous biochar containing graphitic carbon through a two-step activation process. The waste derived carbon was used as adsorbent for the removal of wastewater pharmaceutical pollutants, through an absorption mechanism that changes with pH (Tam et al.). Bagasse and cluster stalks from winemaking waste were transformed into microporous-mesoporous carbon structures through a hydrothermal process followed by alkaline activation. These materials display high electrochemical double-layer capacitance and stability and can be considered for application as negative electrodes in electrochemical energy storage devices (Alcaraz et al.). A detailed knowledge on the physical-chemical and catalytic properties of activated carbons derived from commercial glycerol and of their interaction with reaction reagents and solvents gives an important contribute to the use of biomass derived carbons to produce platform molecules (Tudino et al.).

New materials can be obtained from specific waste-derived precursors, taking advantage of the specific waste features, usually related to the chemical composition of the waste precursor or to its microstructure/morphology. For example, Magnacca et al. evidenced some peculiar properties of porous alumina membranes prepared from bio-based substances from compost, resulting in a selective adsorption of cationic species. In their perspective article, Fiorani et al. describe the chemical complexity of plant-based biomasses, pointing out to the urgency of reliable and convergent chemical strategies for their transformation into platform molecules. As reported by Tummino et al., soybean hulls from agro-industrial scraps can be still used after peroxidase extraction as adsorbents for inorganic and organic pollutants present in wastewater. The authors also evidenced that the adsorption efficiency is strongly dependent on the extraction conditions.

There are plenty opportunities for research work on the chemical synthesis of materials derived from inorganic waste. Therefore, we encourage the scientific community to intensify the research in this specific field, being convinced that any advancement of knowledge in this direction may also have a considerable impact on society. It is enough to consider for example the electric/electronic waste, the waste tires, wastewater, sludges and other types of inorganic industrial waste that are at the moment seldom recycled. Abdelbasir et al. report here a strategy to transform inorganic waste into inorganic nanoparticles for use in environmental applications. However, toxicological and life-cycle aspects should be taken into consideration for upscaling the synthesis process (Abdelbasir et al.). Another strategy is the extraction of metal cations from industrial inorganic waste and their use as inorganic precursors for the synthesis of materials, as described by Tamas et al. for Zn ash used as a Zn precursor. A further step will be to use the inorganic waste directly in the synthesis without any acid/base extraction.

Finally, we would like to thank all authors and reviewers who contributed to this Research Topic. The papers collection under this Research Topic gave a substantial contribution to the knowledge on the use of waste-derived precursors in the synthesis of materials for energy and environmental applications. In the next future, new synthesis procedures need to be explored and greater flexibility will be required to maximize the economic and technological advantages of waste-derived materials production. In this sense, the role of Chemistry as “*a sustainable bridge from waste to materials for energy and environment*” needs to be further consolidated, in order to facilitate the transition from ideas to real industrial-social opportunities.

## Author Contributions

FD wrote the first draft, ET and AT corrected and integrated it.

## Conflict of Interest

The authors declare that the research was conducted in the absence of any commercial or financial relationships that could be construed as a potential conflict of interest.

